# Bioinformatics analysis of ferroptosis-related genes and immune cell infiltration in non-alcoholic fatty liver disease

**DOI:** 10.1186/s40001-023-01457-0

**Published:** 2023-12-19

**Authors:** Huan Zhang, Malina Axinbai, Yuqing Zhao, Jiaoyang Wei, Tongshuo Qu, Jingmin Kong, Yongqiang He, Liping Zhang

**Affiliations:** 1 Department of Digestion, Dongfang Hospital, Beijing University of Chinese Medicine, Beijing, China; 2 Beijing University of Chinese Medicine, Beijing, China; 3 Department of Emergency, Beijing Chaoyang Integrative Medicine Rescue and First Aid Hospital, Beijing, China; 4 Department of Digestion, Dongzhimen Hospital, Beijing University of Chinese Medicine, Beijing, China; 5 Xinjiang Medical University, Urumqi, China; 6 Xiyuan Hospital, China Academy of Chinese Medical Sciences, Beijing, China

**Keywords:** Non-alcoholic fatty liver disease, NAFLD, Ferroptosis, Bioinformatics analysis, Immune

## Abstract

**Background:**

The morbidity and mortality rates of patients with non-alcoholic fatty liver disease (NAFLD) have been steadily increasing in recent years. Previous studies have confirmed the important role of ferroptosis in NAFLD development; however, the precise mechanism through which ferroptosis influences NAFLD occurrence remains unclear. The present study aimed to identify and validate ferroptosis-related genes involved in NAFLD pathogenesis and to investigate the underlying molecular mechanisms of NAFLD.

**Methods:**

We downloaded microarray datasets GSE72756 and GSE24807 to identify differentially expressed genes (DEGs) between samples from healthy individuals and patients with NAFLD. From these DEGs, we extracted ferroptosis-related DEGs. GSE89632, another microarray dataset, was used to validate the expression of ferroptosis-related genes. A protein–protein interaction (PPI) network of ferroptosis-related genes was then constructed. The target genes were also subjected to Gene Ontology (GO) and Kyoto Encyclopedia of Genes and Genomes (KEGG) pathway enrichment analyses. Finally, competing endogenous RNA networks were constructed. We used the CIBERSORT package to evaluate the infiltration of immune cells infiltration in NAFLD.

**Results:**

Five ferroptosis-related genes (*SCP2*, *MUC1, DPP4, SLC1A4,* and *TF*) were identified as promising diagnostic biomarkers for NAFLD. Enrichment analyses revealed that these genes are mainly involved in metabolic processes. NEAT1-miR-1224-5p-SCP2, NEAT1-miR-485-5p-MUC1, MALAT1-miR-485-5p-MUC1, and CNOT6-miR-145-5p-SLC1A4 are likely to be the potential RNA regulatory pathways that affect NAFLD development. Principal component analysis indicated significant differences in immune cell infiltration between the two groups.

**Conclusions:**

This study identified five ferroptosis-related genes as potential biomarkers for diagnosing NAFLD. The correlations between the expression of ferroptosis-related genes and immune cell infiltration might shed light on the study of the molecular mechanism underlying NAFLD development.

**Supplementary Information:**

The online version contains supplementary material available at 10.1186/s40001-023-01457-0.

## Introduction

Non-alcoholic fatty liver disease (NAFLD) is an important liver disease that affects approximately 24% of the general population [1]. In the coming decades, NAFLD might become the leading cause of end-stage liver disease [1]. NAFLD encompasses a range of diseases from non-alcoholic fatty liver (NAFL) to non-alcoholic steatohepatitis (NASH), fibrosis, and cirrhosis [2]. An underlying progressive liver disease is typically observed in a subset of patients with NAFLD..

NAFLD is usually diagnosed by an invasive liver biopsy. Presently, there are no reliable biomarkers for accurately diagnosing and staging NAFLD, which makes it challenging to screen NAFLD cases worldwide [3]. Moreover, according to the current hypothesis, NAFLD is the hepatic manifestation of metabolic syndrome because of its bidirectional association with the components of metabolic syndrome [3, 4]. NAFLD patients show a high incidence rate of metabolic complications; hence, NAFLD is considered a growing burden on the healthcare system [5]. Therefore, it is crucial to identify new and efficient NAFLD biomarkers for the prompt diagnosis and treatment of this disease.

Ferroptosis is an iron-dependent form of programmed cell death. It is characterized by the cellular accumulation of lipid hydroperoxides to lethal levels [6]. The morphological effects of ferroptosis include reduced mitochondrial size, disappearance of mitochondrial cristae, and mitochondrial membrane rupture [7]. The primary changes in biochemical characteristics associated with ferroptosis are iron overload and decreased glutathione peroxidase 4 (GPX4) activity; these changes promote the production of reactive oxygen species (ROS), accelerate lipid peroxidation, and eventually lead to cell death [8]. Ferroptosis is associated with the onset and progression of many liver diseases such as NAFLD, alcohol-associated liver disease (ALD), hepatocellular carcinoma (HCC), and hepatitis C virus (HCV) infection [9–13]. Alterations in several metabolic pathways, including decreased GPX4 activity, iron overload, acyl-CoA synthetase long-chain family member 4 (ACSL4) induction, and nuclear factor erythroid-2-related factor 2 (Nrf2) activation, have been implicated in ferroptosis [14, 15]. Therefore, ferroptosis inhibition could serve as a new treatment approach for NAFLD. It, however, remains unclear how ferroptosis regulates NAFLD.

In the study, we analyzed two NAFLD liver tissue-derived microarray datasets from the Gene Expression Omnibus (GEO) database and obtained differentially expressed genes (DEGs). From these DEGs, we selected ferroptosis-related genes (FRGs). The expression of these FRGs was further validated in another microarray dataset. Finally, five genes, namely *SCP2*, *MUC1, DPP4, SLC1A4,* and *TF*, were screened as target genes. Competing endogenous RNA (ceRNA) networks were constructed to determine the specific regulatory effects of noncoding RNAs on the FRGs in NAFLD. The ratios of immune cell infiltration in NAFLD and normal tissues were calculated using the CIBERSORT package. We also evaluated the correlations between the expression of FRGs and infiltration ratios of various immune cells. This in-depth research investigated the mechanism of NAFLD development at the transcriptome level and identified potential biomarkers for NAFLD diagnosis.

## Materials and methods

### Search strategy

We searched for NAFLD-associated gene expression microarrays in the GEO database (https://www.ncbi.nlm.nih.gov/geo/). The screening criteria were as follows: (1) the biological type was restricted to *Homo sapiens*, (2) liver tissues were obtained exclusively from patients with NAFLD or NASH, (3) the number of samples in each dataset exceeded five, (4) complete information of the samples was available, and (5) each sample was analyzed only once without replication. Finally, GSE72756 and GSE24807 datasets were selected as the test datasets and included 5 NAFLD samples and 12 NASH samples, respectively (Additional file 1: Table S1). The GSE89632 dataset was used as the validation dataset and included 39 NAFLD samples (20 simple steatosis samples and 19 NASH samples) and 24 healthy samples. A list of all FRGs (259 genes) was collected from the FerrDb database (http://www.zhounan.org/ferrdb/).

### Microarray data

GSE72756 includes the expression data for 5 normal and 5 NAFLD liver samples. GSE24807 contains the expression data for 5 normal and 12 NASH liver samples. The microarray platforms and a series of matrix files downloaded from the GEO database were saved as TXT files.

### Identification of FRGs

R software (version 4.1.3) was used to process the two raw datasets. The microarray platforms and the series of matrix files were converted into annotation packages (https://bioconductor.org/biocLite.R). The microarray datasets were quantile-normalized by the limma package of R software [16] and saved as a TXT file. We used the linear model and the empirical Bayes test from the limma package to filter the expression profile data and to screen DEGs. The screening criteria for DEGs were as follows: log2 (fold change) > 1 and *p*-value < 0.05. A volcano plot and a heatmap were constructed using R software to better visualize DEGs. The volcano plot was created using the “ggplot2” package [17]. The heatmap was drawn using the heatmap package [18]. To identify FRGs, we created a Venn map using the Venn tool (http://bioinformatics.psb.ugent.be/webtools/Venn/), and the overlapping DEGs were retained for further analysis.

### Validation of the FRGs

We verified the expression of the FRGs in the GSE89632 dataset. To assess the sensitivity and specificity of the selected FRGs for NAFLD diagnosis, the visualization tool Hiplot(https://hiplot.com.cn) was used to generate a receiver operating characteristic (ROC) curve. We evaluated the performance of each model by calculating the area under the ROC curve (AUC) value; an AUC value of > 0.6 was considered statistically significant.

### Construction of a protein–protein interaction network

The STRING online tool (https://string-db.org/) was used to construct a protein–protein interaction (PPI) network of the obtained DEGs [19]. The PPI network of the DEGs was constructed based on the confidence score of 0.4. Next, the STRING analysis data and the FRGs were plotted using the Cytoscape software (version 3.7.2) for better visualization.

### Functional enrichment analysis of the FRGs

We conducted Gene Ontology (GO) enrichment analyses of the FRGs by using packages such as “ggplot2” and “cluster profile”. The three criteria for the enriched GO terms included: biological process (BP), cellular component (CC), and molecular function (MF). Kyoto Encyclopedia of Gene and Genomes (KEGG) pathway enrichment analysis was conducted using an online platform (http://www.bioinformatics.com.cn/) that analyzes and visualizes data [20].

### Construction of ceRNA networks

We predicted interactions between ferroptosis-related mRNAs and miRNAs by using five online miRNA databases: miRWalk, TargetScan, DIANA, PITA, and miRanda. As target miRNAs, we identified miRNAs predicted in at least three of these five databases. Based on the selection of miRNAs, we also predicted lncRNAs and circRNAs that interact with these miRNAs by using StarBase (version 3.0; http://starbase.sysu.edu.cn/index.php). The OmicShare tools (https://www.omicshare.com/tools/Home) were used to visualize the ceRNA networks [21].

### Immune cell infiltration analysis

The abundance and difference in immune cell infiltration were assessed between NAFLD and healthy liver tissues. We used the CIBERSORT algorithm to analyze the infiltration of 22 types of immune cells [22]. A correlation heatmap was drawn using the “corrplot” package [23] to demonstrate the association between the 22 types of immune cells. The results of principal component analysis (PCA) were plotted using the “ggplot2” package [17] to determine the differences between NAFLD samples and normal samples. The Wilcoxon test was used to identify significantly differential immune cell infiltration in the model and control groups. The R packages “vioplot”, “ggplot2”, and “glment” [24] were used to show differences in the level of immune cell infiltration in the two groups. Finally, we conducted Spearman’s rank correlation analysis in R software to determine the association between the FRGs and the immune cells infiltration level.

## Results

### Identification and analysis of the DEGs

**Fig. 1:**
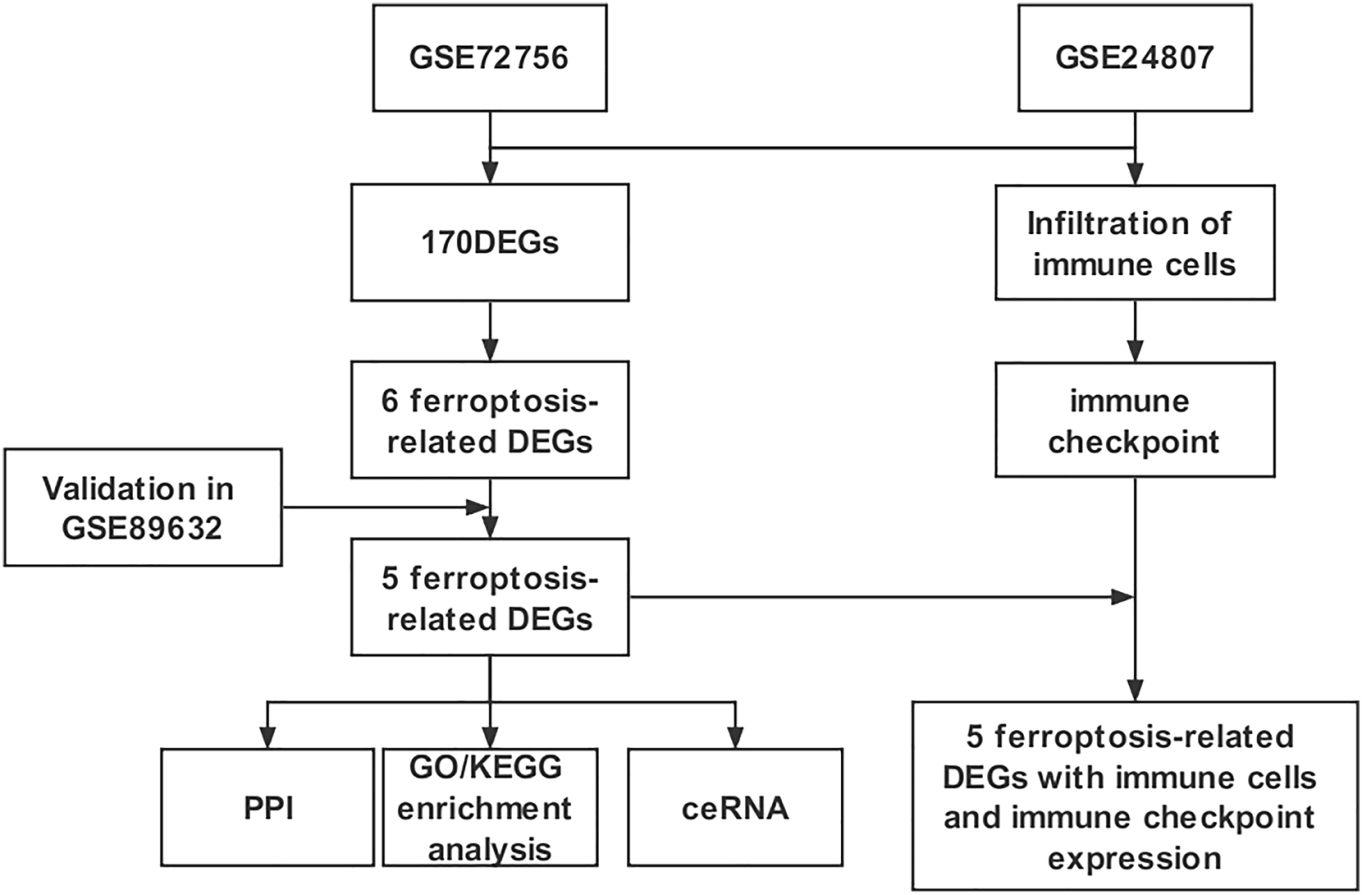
Study flowchart

**Fig. 2:**
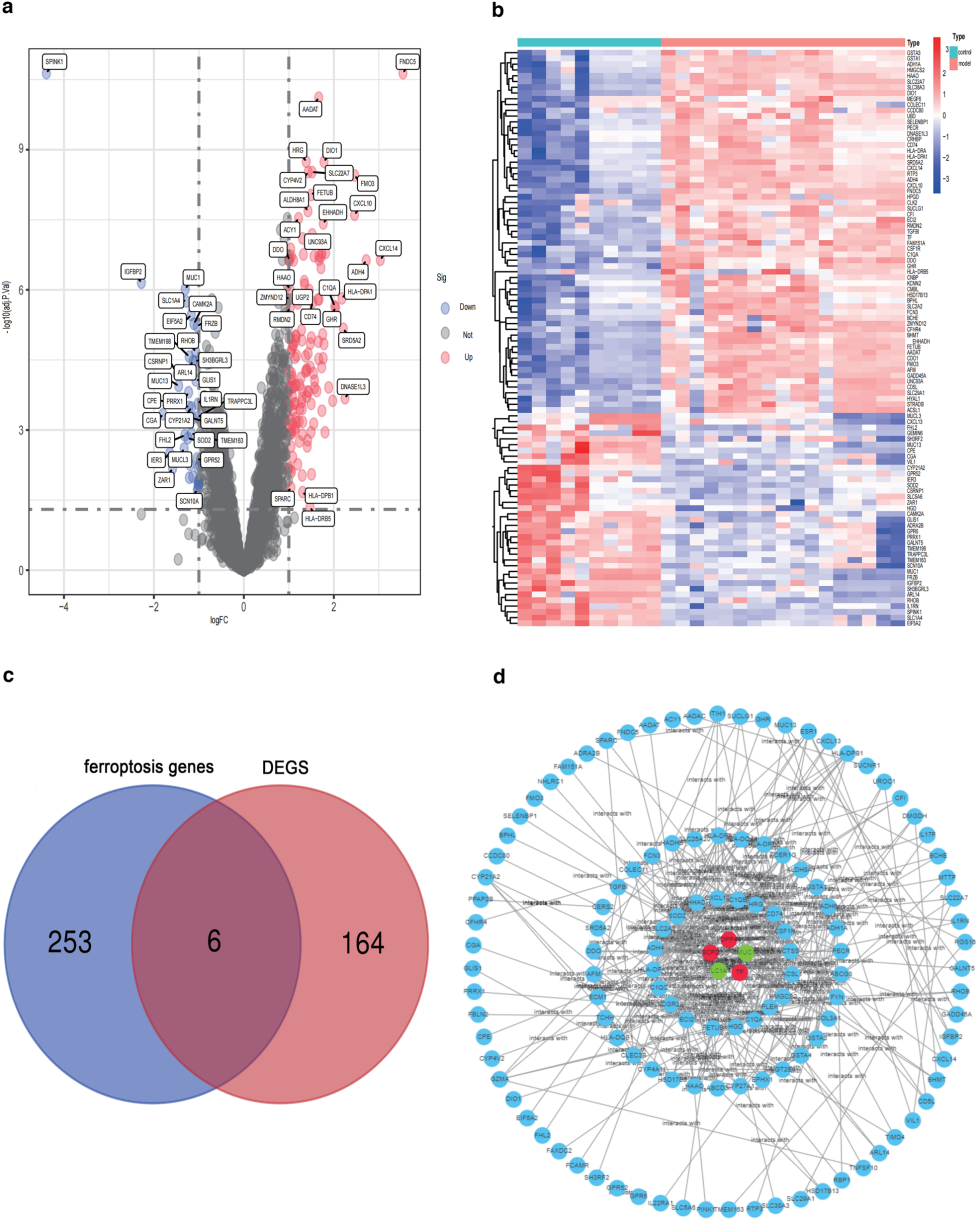
DEGs volcano map and heat map between NAFLD group and control group. **a** DEGs volcano plot. The blue plots represent downregulated genes, the red plots represent upregulated genes, and the grey plots represent nonsignificant genes. **b** DEGs heat map, blue dots represent downregulated genes, while red dots represent upregulated genes. **c** Ferroptosis-related genes. **d** PPI network. Blue represents the DGEs that are not associated with ferroptosis. The red indicates the upregulated FRGs and the green indicates the downregulated. The line indicates the interaction between two proteins

We selected GSE72756 and GSE24807 datasets to analyze and identify the DEGs. Figure 1 shows the schematic flowchart of the analysis. A total of 170 DEGs were identified, which include 133 upregulated DEGs and 37 downregulated DEGs (Additional file 2: Table S2). The volcano plot and heatmap are shown in Fig.2a, b. Based on the Venn diagram, the following genes were identified to be associated with ferroptosis: cysteine dioxygenase 1 (*CDO1*), dipeptidyl peptidase-4 (*DPP4*), solute carrier family 1 member 4 (*SLC1A4*), mucin 1 (MUC1), sterol carrier protein 2 (*SCP2*), and tissue factor (*TF*) (Fig. 2c). We then generated PPI correlation networks associated with these FRGs to clarify their transcriptomic characteristics (Fig. 2d). Among the identified FRGs, *CDO1* showed no PPI with the other DEGs.

### Confirmation of the expression and diagnostic value of the FRGs using the GSE89632 dataset

**Fig. 3:**
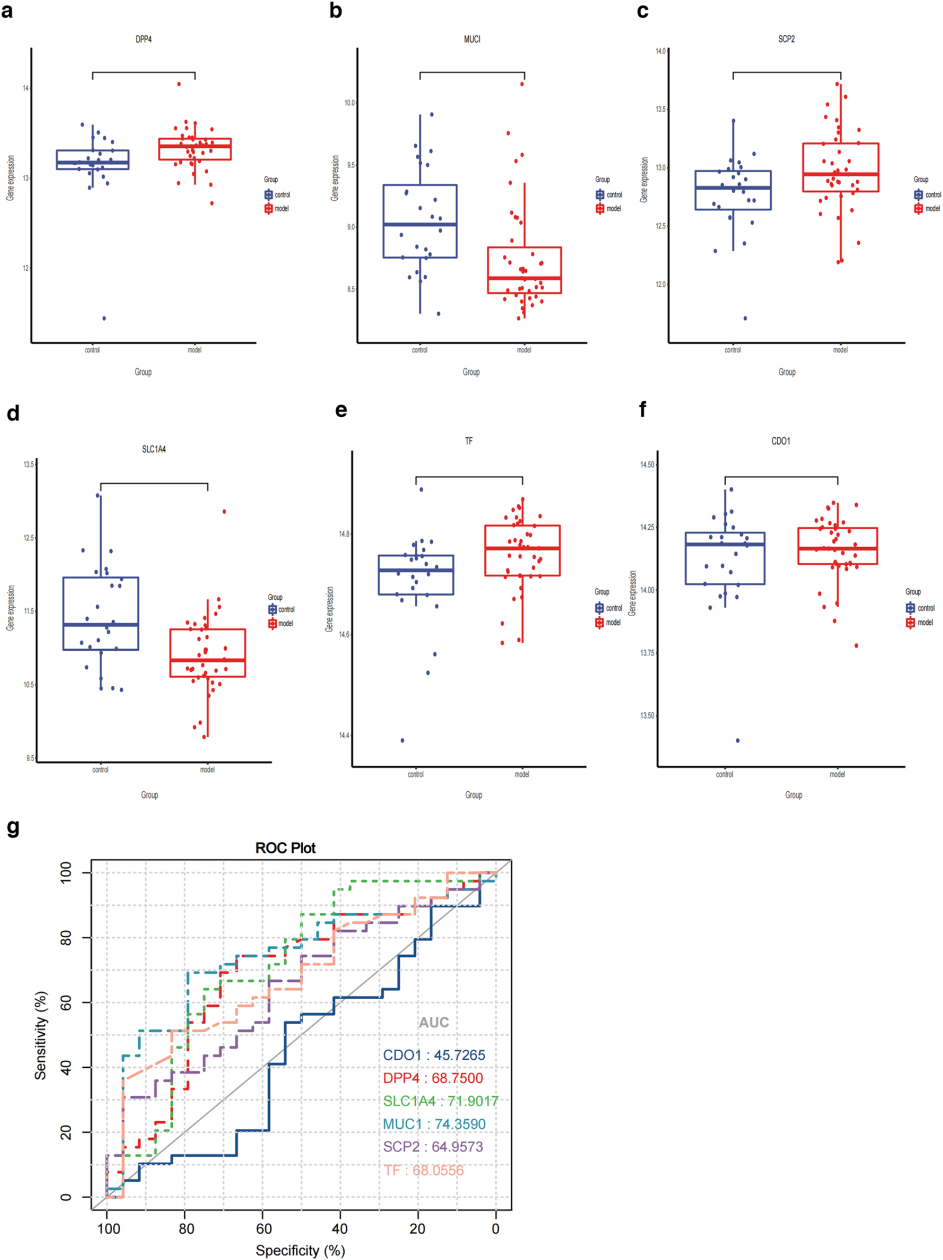
Six ferroptosis-related genes expression and diagnostic performance in GSE89632. **a**–**f** The expression of six ferroptosis-related genes in NAFLD samples and healthy samples. **g** Diagnosis efficiency ROC curve of the feature genes of six ferroptosis-related genes in NAFLD and normal samples

Because the original dataset from the GEO database might have been processed by the original authors, which may be unknown, we used an independent external dataset to further validate and confirm the relevance of our results. We tested the expression levels of our screened target genes by using the GSE89632 dataset. Consistent with the predicted results, significant differences were observed in the expression levels of the above-mentioned five FRGs (*DPP4, MUC1, SCP2, SLC1A4,* and *TF*.) between NAFLD patients and healthy subjects (Fig. 3a–e). However, the expression of CDO1 did not differ significantly between the two groups (Fig. 3f). To determine the most significant FRGs for diagnosing NAFLD, we further performed ROC curve analysis by using the GSE89632 dataset. As shown in Fig. 3g, *SLC1A4 *and *MUC1 *(AUC = 0.719 and 0.744, respectively) had a credible diagnostic value for NAFLD. *DPP4*, *SCP2*, and *TF*(AUC = 0.688, 0.650, 0.681, respectively) showed a tolerable ability to distinguish NAFLD samples from normal samples.

### Pathways enrichment analysis of the FRGs

**Fig. 4:**
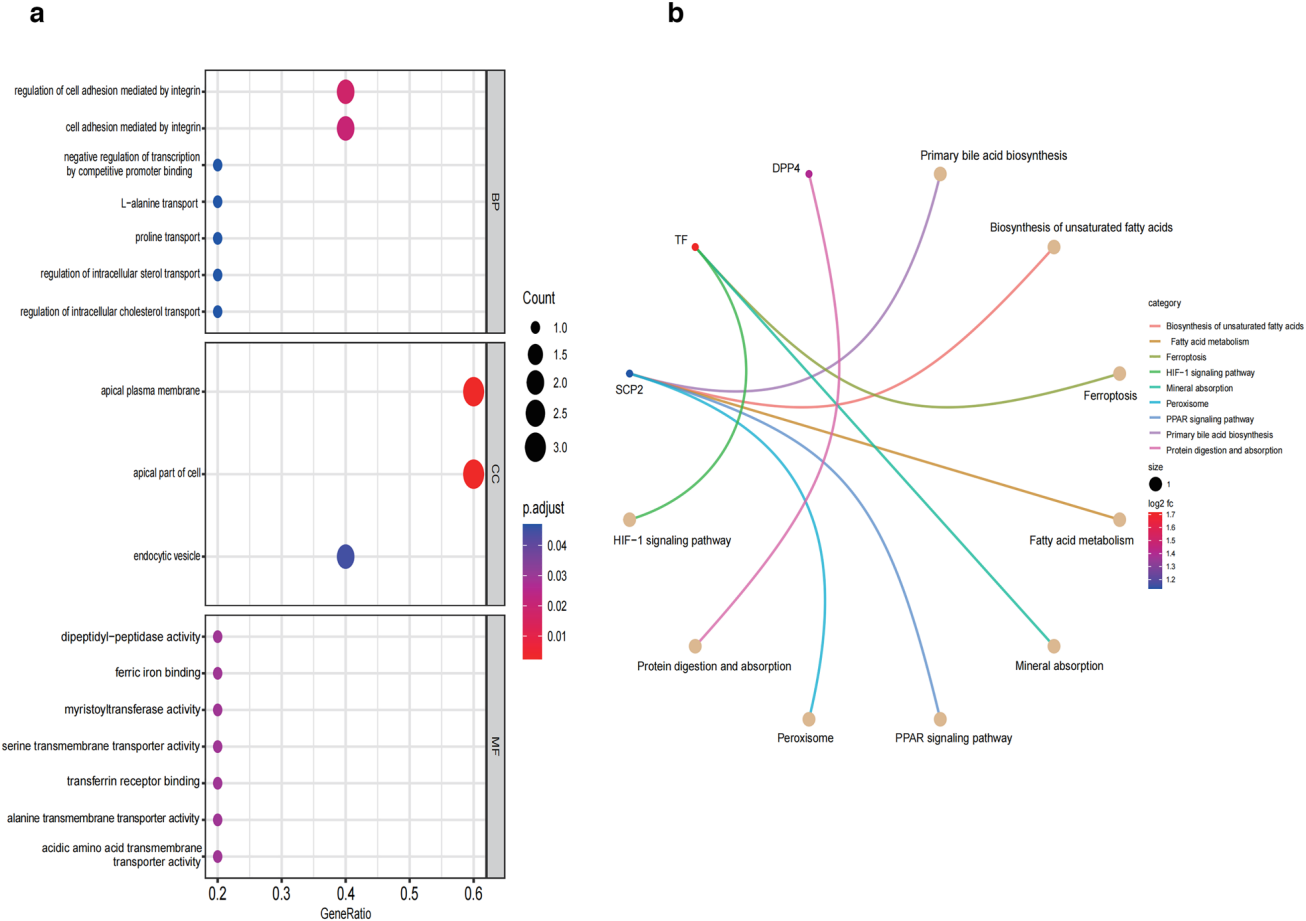
GO and KEGG pathway enrichment of five ferroptosis-related genes. **a** GO analysis. *BP* biological process, *CC* cellular component, *MF* molecular function. **b** KEGG pathway enrichment analysis. Count indicates the level of enrichment. Different colors represent the *p*-value

The GO enrichment analysis revealed the following most GO significant enrichment terms: (1) BP: regulation of intracellular cholesterol and lipid transport, organic acid biosynthetic and catabolic process, regulation of cell adhesion mediated by integrin, fatty acid beta-oxidation using acyl-CoA oxidase, and regulation of iron ion transport; (2) CC: apical plasma membrane, endocytic vesicle, and apical part of the cell; and (3) MF: fatty acid binding, cholesterol-binding, ferric iron binding, transferrin receptor binding, and acidic amino acid transmembrane transporter activity (Fig. 4a, Additional file 3: Table S3). The KEGG pathway enrichment analysis revealed that the FRGs were particularly enriched in metabolic processes such as biosynthesis of unsaturated fatty acids, fatty acid metabolism, primary bile acid biosynthesis, and peroxisome and HIF-1 signaling pathway (Fig. 4b, Additional file 4: Table S4).

### Construction of ceRNA networks

**Fig. 5:**
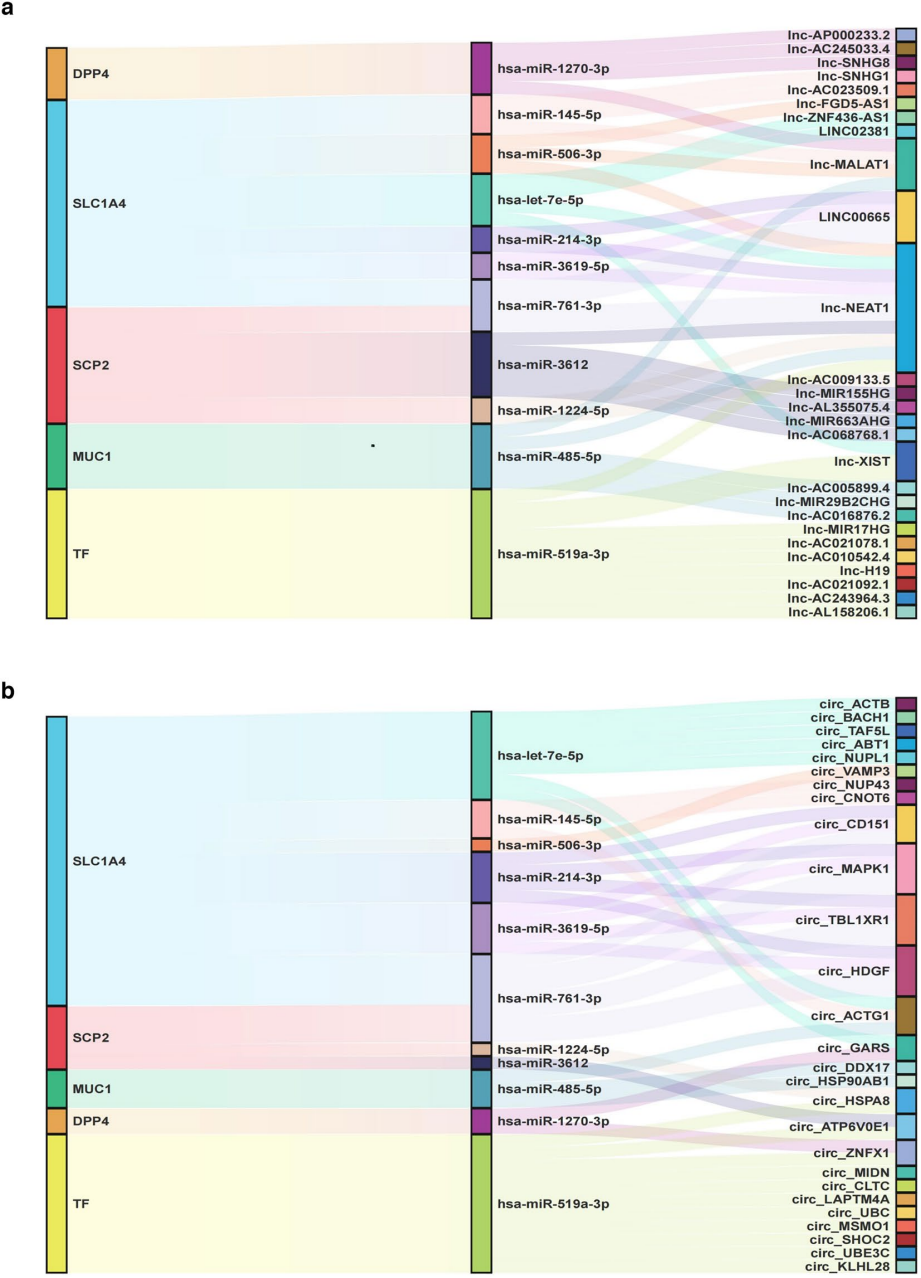
The Sankey diagram describes five ferroptosis-related genes in the ceRNA network. **a** lncRNA–miRNA–mRNA network, **b** circRNA–miRNA–mRNA network

We predicted the target miRNAs of the five FRGs by using five online miRNA databases and determined 11 target miRNAs and 12 mRNA–miRNA pairs (Additional file 1, Additional file 5: Table S5). Subsequently, we predicted circRNA and lncRNA mediated effects on the selected miRNAs by using the online database Starbase 3.0. We selected circRNAs and lncRNAs associated with the largest number of database samples and the highest scores based on the CLIP-seq data and clipExpNum (Additional file 6: Tables S6 and Additional file 7: Table S7). Finally, we constructed two ceRNA networks and obtained 42 lncRNA–miRNA pairs and 41 circRNA–miRNA pairs related to the five target genes (Fig. 5a, b).

### Immune cell infiltration analysis

**Fig. 6:**
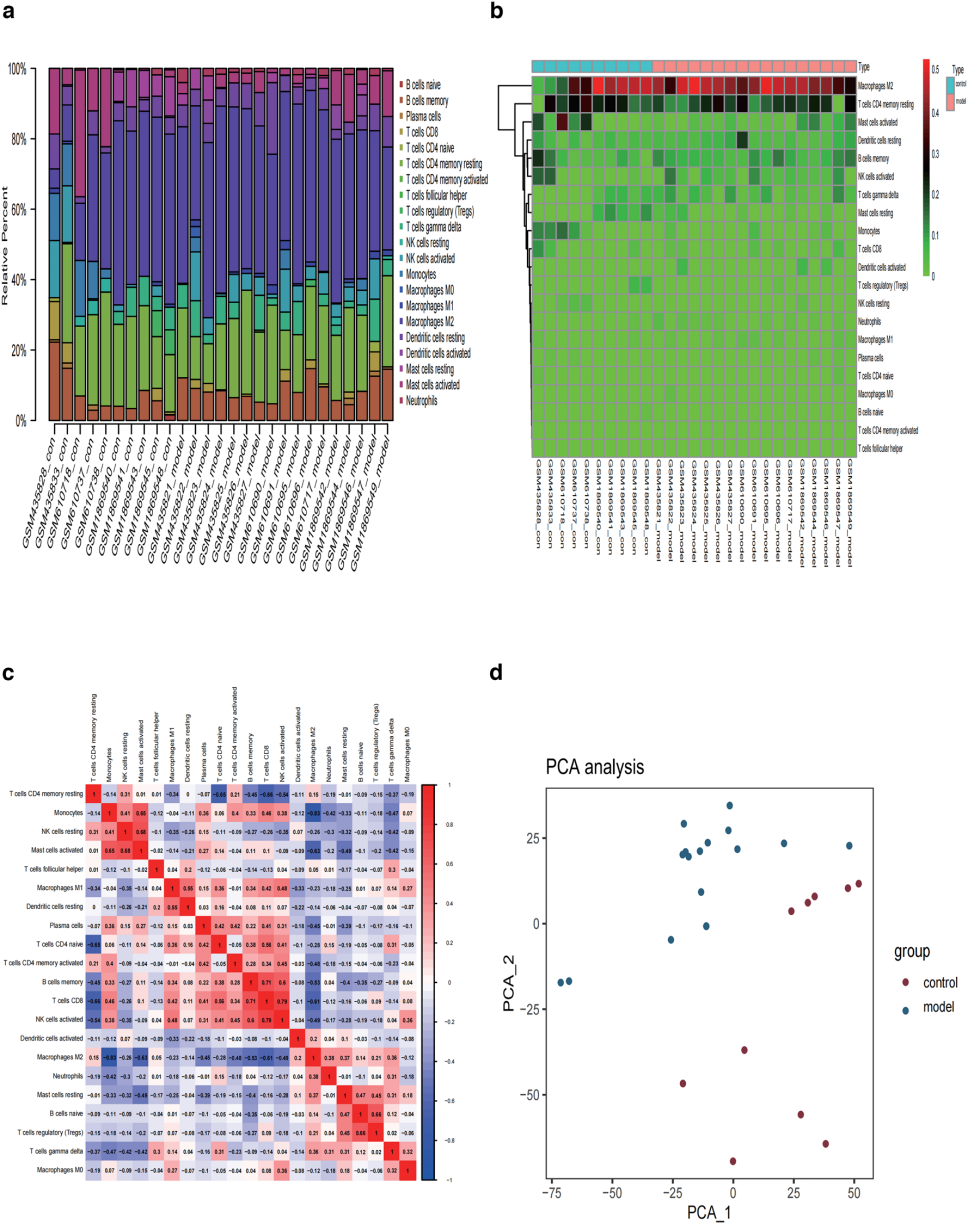
The situation of immune infiltration in liver tissues. **a** The histogram displays the relative percentage of immune cells in each sample, with different colors representing different immune cells. **b** The heatmap represents the expression levels of immune cells in each sample. Red indicates higher immune infiltration expression, while green indicates lower expression. **c** Correlation of the 22 immune cells. Red: positive correlation; blue: negative correlation. **d** Two groups of samples were analyzed using PCA: blue points indicate NAFLD samples and red points indicate normal samples

The composition of the 22 types of immune cells in each sample was quantified using the CIBERSORT algorithm (Fig. 6a, b). The results showed that M2 macrophages, CD4^+^ memory resting T cells, activated mast cells, memory B cells, and activated NK cells were the main immune infiltrating cells. The correlation of the 22 types of immune cells is shown in Fig. 6c. M2 macrophages showed a positive correlation with monocytes, activated mast cells, and CD8^+^ T cells. CD8^+^ T cells were positively correlated with activated NK-cell. PCA of NAFLD patients and healthy subjects showed no intersection of the two clusters, thus indicating a significant difference in the level of immune cell infiltration between the two groups (Fig. 6d, Additional file 8: Table S8).

**Fig. 7:**
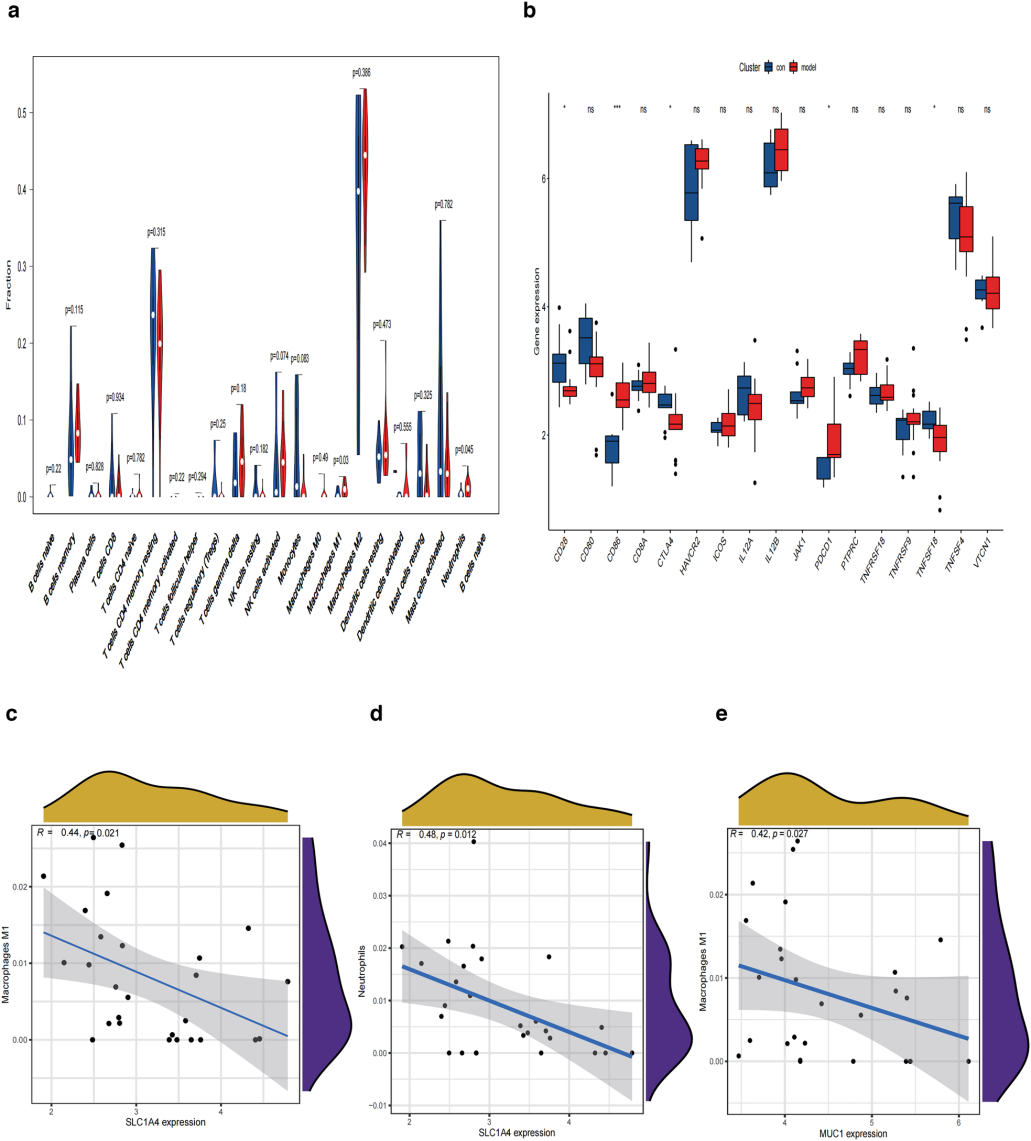
Immune infiltration in NAFLD and normal controls. **a** Violin diagram of the proportion of 22 types of immune cells. **b** Box plots of immune checkpoints in the two groups. *P < 0.05, **P < 0.01, ***P < 0.001. **c**–**e** The correlation between ferroptosis-related DEGs and immune cells

We then conducted a cellular and molecular level analysis to further elucidate the relationship between the FRGs and the immune profile of the NAFLD group. The analysis revealed a higher fraction of immune cell infiltration in the NAFLD group, including infiltration of immune cells such as M1 macrophages and neutrophils and expression of the immune checkpoint markers such as CD86 and PDCD1 (Fig. 7a, b). We then investigated the relationship between the expression levels of the five FRGs with the infiltrating levels of M1 macrophages and neutrophils. Because multiple tests were conducted, we applied the Bonferroni correction for multiple test [25]. We tested the correlation between the FRGs and two types of immune cells. Therefore, the significance level of *P* = 0.05 was divided by 2, resulting in a significance level of *P* = 0.025 after correction for multiple testing. As shown in Fig. 7c–e M1 macrophage activation was negatively correlated with *MUC1* and *SLC1A4*, and neutrophil activation was negatively correlated with *SLC1A4* (*R* < − 0.40, *p* < 0.025).

## Discussion

NAFLD is the most common chronic liver disease worldwide. Ferroptosis plays a critical role in the occurrence and progression of NAFLD through the regulation of iron homeostasis and lipid metabolism in the liver. Hence, the identification of new effective NAFLD biomarkers could enable prompt diagnosis timely and treatment of this disease.

In the present study, we attempted to identify ferroptosis-related biomarkers in NAFLD and examined the role of immune cell infiltration in NAFLD pathogenesis. Six FRGs were identified using two liver tissue microarray datasets (GSE72756 and GSE24807) from the GEO database and the FerrDb database. Of these, five FRGs (*SCP2, MUC1, DPP4, SLC1A4,* and *TF*) were finally identified after confirmation using the GSE89632 dataset. The pathway enrichment analysis revealed that these genes were primarily involved in metabolic processes. The most enriched GO categories were regulation of intracellular cholesterol and lipid transport, regulation of intracellular cholesterol and lipid transport, organic acid biosynthetic and catabolic process, fatty acid beta-oxidation using acyl-CoA oxidase, cholesterol-binding, fatty acid-binding, and acidic amino acid transmembrane transporter activity. The KEGG pathway enrichment analyses showed that the main functions of these genes were the biosynthesis and metabolism of fatty acids, primary bile acid biosynthesis, protein digestion and absorption, peroxisome, and regulation of the HIF-1 signaling pathway and the PPAR signaling pathway.

Among the five selected FRGs, *SCP2* and *DPP4* are ferroptosis driver genes, *MUC1* is a ferroptosis suppressor gene, *SLC1A4* is a ferroptosis marker gene, and *TF* is a ferroptosis driver and marker gene [26]. We focused on two genes: *SCP2* related to lipid metabolism and *MUC1* with the highest diagnostic specificity. The lipid transport protein *SCP2* can combine with fatty acids and fatty acyl-COA to regulate fatty acid metabolism in the liver [27, 28]. It is also a key regulator of cholesterol metabolism in the liver and plays a beneficial role in NAFLD. In contrast to the protective effect of *SCP2* on NAFLD, a previous study showed that *SCP2* can promote the accumulation of low-density lipoprotein cholesterol (LDL-C), thereby promoting the development of atherosclerosis and hyperlipidemia [29]. Previous studies have indicated that *SCP2* can suppress ferroptosis inhibitors (GPX4 and cav1) and activate ferroptosis promoters (PRKAA1 and PRKAA2) [30]. *MUC1* is a large O-type glycoprotein essential for maintaining the function of the epithelial cell surface [31]. It is composed of two subunits: the *MUC1* N-terminal subunit (MUC1-n) and the carcinogenic *MUC1* C-terminal subunit (MUC1-c) that form a heterogeneous complex on the cell membrane [32]. Many MUC1-c subunits can be detected in the mitochondria and nuclei of cancer cells. Several studies have shown that *MUC1* plays a key regulatory role in tumor invasion, metastasis, angiogenesis, and inflammation [33–36]. *MUC1* can also induce apoptosis and necrosis by inhibiting ROS accumulation [37]. Hasegawa et al. demonstrated that targeting MUC1-c with ferroptosis inhibitors induces ROS-mediated death [38].

Yangchunxie et al. confirmed that *DPP4* (also known as CD26) plays a role in ferroptosis regulation and found that the loss of TP53 prevented the nuclear accumulation of *DPP4* in colorectal cancer cells, thereby facilitating the plasma membrane-related DPP4-dependent lipid peroxidation and ultimately leading to ferroptosis [39]. These data support *DPP4* as the driving factor of ferroptosis. *TF* is an extremely important factor in regulating iron trafficking and metabolism. The increased expression of *TF* is suggested to induce ferroptosis [40]. *SLC1A4* is one of the members of solute carrier family 1, and it can promote ferroptosis [41]. It should be noted that most of these aforementioned genes have been identified in tumors; however, there is a general lack of evidence regarding their role in NAFLD.

In the present study, two ceRNA networks were constructed to determine the regulatory mechanisms of these five FRGs by predicting their miRNA targets as well as the lncRNAs and circRNAs targeted by these miRNAs. According to the ceRNA hypothesis, we searched literature related to NAFLD in the PubMed database and selected 3 reported miRNAs, 2 lncRNAs, and 1 circRNA for further investigation. Based on our findings, we suggest that MALAT1-miR-485-5p-MUC1, NEAT1-miR-1224-5p-SCP2, and NEAT1-miR-485-5p-MUC1 might be the regulatory pathways for the pathogenesis and progression of NAFLD. MALAT1 and NEAT1 are important lncRNAs, and recent studies have reported that their expression is upregulated in the liver tissues of NAFLD patients. MALAT1 knockdown reversed free fatty acid -induced lipid accumulation in hepatocytes; moreover, MALAT1 promoted the progression of liver fibrosis [42]. NEAT1 was previously identified as an oncogene that promotes tumor cell proliferation [43]. Several recent studies have shown that NEAT1 participates in NAFLD progression by promoting lipid deposition in the liver [44]. The regulatory relationship between NEAT1 and ferroptosis has been reported in recent literature. Zhang et al. discovered that NEAT1 overexpression enhances both extracellular and intracellular ferroptosis, thereby increasing the anti-tumor activity of erastin [45]. miR-1224-5p promotes hepatic lipogenesis by inhibiting AMPKα1 expression [46]. miR-1224-5p inhibitors deserve further investigation as a potential therapeutic tool for treating NAFLD. miR-485-5p is associated with inflammation and immune responses, and it upregulates *MUC1* to promote liver cancer progression [47]. MALAT1 and NEAT1 target miRNAs in NAFLD to regulate ferroptosis; this aspect requires further investigation. Regarding circRNAs, although circ_CNOT6 has not been reported in NAFLD, it is likely to play a critical role in other metabolic diseases such as diabetes [48]. Therefore, we hypothesized that circ_CNOT6-miR-145-5p-SLC1A4 might be involved in NAFLD development. Further prospective cohort studies are required to confirm our hypothesis.

The primary cause of NAFLD is metabolic dysfunction. Immune cell-mediated inflammatory processes also contribute to NAFLD. The liver immune cell landscape directly affects the severity of NAFLD. A study conducted by the German Cancer Research Center showed the accumulation of a large number of CD8/PD-1 double-positive abnormal T cells in the liver of NASH patients. PD-1/L1 inhibitors can activate these T cells; however, treatment with PD-1/L1 inhibitors not only kill the tumor cells but also aggravates liver tissue damage [49]. Previous studies have confirmed that immunotherapy has no survival benefit for liver cancer patients with NAFLD.

To better understand immune cell infiltration, we used the CIBERSORT algorithm to evaluate immune cell infiltration in NAFLD tissues. We found that increased infiltration of M1 macrophages and neutrophil was associated with NAFLD occurrence and development. We also found that the M1 macrophage activation was negatively correlated with *MUC1* and *SLC1A4* and neutrophil activation was negatively correlated with *SLC1A4*.

According to previous studies, the beneficial effects of neutrophils during infections are opposite to those in noninfectious diseases. Neutrophils usually produce neutrophil extracellular traps, proteases, cytokines, and ROS to induce adverse effects on the infectious agent [50, 51]. Several studies have reported significantly increased neutrophil infiltration in the liver of patients with NASH [52, 53]. Neutrophils are involved in the early stages of NASH development. However, their role in the advanced stage of NASH remains unclear [54]. In an in vivo study, Zhao et al. confirmed that methionine-choline-deficient and high-fat (MCDHF) diet-induced liver injury was significantly reduced by an intraperitoneal injection of deoxyribonuclease I [53]. Blood monocytes are recruited to hepatic sinusoids and differentiate into macrophages, thereby increasing the macrophage pool of the liver [55]. Recent studies have shown that monocyte-derived macrophages exhibit more apparent inflammatory characteristics in NASH and can promote injury by limiting liver lipid storage in the liver [56]. Monocyte-derived macrophages in mouse livers are located in the tissue fibrosis area near desmin-positive hepatic stellate cells, thus indicating their contribution to liver fibrosis [57]. These studies and our present analysis support the concept that immune cell infiltration is an important factor in NAFLD pathogenesis. Future studies should focus on the correlation between FGRs and M1 macrophages and neutrophils.

In the present study, we identified DEGs associated with ferroptosis in NAFLD. Our findings also suggest a certain correlation between FRGs and immune cell infiltration in NAFLD. Furthermore, we identified NAFLD-related miRNAs, lncRNAs, and circRNAs. However, because this was a strictly bioinformatics analysis, in future studies, we will focus on the expression patterns and functions of these genes to understand the precise molecular mechanism of ferroptosis in NAFLD development. First, we will quantify the expression of these genes at the transcriptional and translational levels and confirm their interactions through immunohistochemical and immunofluorescence assays. Second, we will determine the specific mechanisms of ferroptosis in lipid accumulation, hepatocyte injury, and immune responses by using cellular models. Third, we will collect more liver tissue samples from NAFLD patients for conducting large-scale research. Our research will focus on identifying more effective ferroptosis-specific biomarkers and developing ferroptosis modulators with improved properties for alleviating NAFLD.

The present study has several limitations. First, we did not perform an additional in vivo experiment to validate whether the selected FRGs regulate ferroptosis in NAFLD. Second, we are aware of FRGs only from the FerrDb database, and only a few studies have examined the role of ferroptosis in NAFLD. Third, we did not evaluate the different stages of NAFLD. To overcome these limitations, prospective clinical trials should be designed to elucidate the mechanisms of action of the five FRGs in different stages of NAFLD.

## Conclusions

In summary, based on our bioinformatics analysis, we identified five FRGs (*SCP2, MUC1, DPP4, SLC1A4,* and *TF*) that could predict NAFLD development and explored the potential pathway of liver tissue damage in NAFLD. We also provided new insights into the molecular mechanisms of NAFLD pathogenesis. Further research is required to confirm our preliminary evidence and to validate these FRGs as proposed biomarkers for NAFLD diagnosis in clinical practice.

### Supplementary Information

**Additional file 1: Table S1.** Clinical information for the datasets.

**Additional file 2: Table S2.** DEGs in the database.

**Additional file 3: Table S3.** Go analysis results.

**Additional file 4: Table S4.** KEGG pathway results.

**Additional file 5: Table S5.** miRNAs interact with mRNAs.

**Additional file 6: Table S6.** miRNAs interact with lincRNAs.

**Additional file 7: Table S7.** miRNAs interact with circRNAs.

**Additional file 8: Table S8. **Result of immune cell infiltration.

## Data Availability

The following information on data availability is provided: raw measurements are provided in the Supplementary file. Further inquiries can be directed to the corresponding authors.

## Abbreviations

NAFLD: Non-alcoholic fatty liver disease
DEGs: Differentially expressed genes
PPI: Protein–protein interaction
GO: Gene Ontology
KEGG: Kyoto Encyclopedia of Genes and Genomes
PCA: Principal component analysis
NAFL: Non-alcoholic fatty liver
NASH: Non-alcoholic steatohepatitis
GPX4: Glutathione peroxidase 4
ROS: Reactive oxygen species
ALD: Alcohol-associated liver disease
HCC: Hepatocellular carcinoma
HCV: Hepatitis C virus
ACSL4: Acyl-CoA synthetase long-chain family member 4
Nrf2: Nuclear factor-E2-related factor 2
GEO: Gene Expression Omnibus
FRGs: Ferroptosis-related genes
ROC: Receiver operating characteristic
AUC: Area under the ROC curve
BP: Biological process
CC: Cell component
MF: Molecular function
CDO1: Cysteine dioxygenase 1
DPP4: Dipeptidyl peptidase-4
SLC1A4: Solute carrier family 1 member 4
MUC1: Mucin 1
SCP2: Sterol carrier protein 2
TF: Tissue factor
LDL-C: Low-density lipoprotein cholesterol
HDL-C: High-density lipoprotein cholesterol
MCDHF: Methionine-choline-deficient and high-fat
