# Supplementary material for: Bioinformatics analysis of ferroptosis-related genes and immune cell infiltration in non-alcoholic fatty liver disease
Source: Eur J Med Res. 2023 Dec 19;28:605. doi: 10.1186/s40001-023-01457-0 (PMC10729346; doi:10.1186/s40001-023-01457-0)
Supplement: Supplementary file 5 — Additional file 5: Table S5. miRNAs interact with mRNAs. [file 40001_2023_1457_MOESM5_ESM.doc]

**Table S5 . miRNAs interact with mRNAs**

| **geneName** | **miRNAname** | **MiRwalk** | **Target scan** | **DIANA** | **PITA** | **miRanda** |
| --- | --- | --- | --- | --- | --- | --- |
| **DPP4** | **hsa-miR-1270-3p** | **1** | **1** | **1** | **0** | **0** |
| **SLC1A4** | **hsa-let-7e-5p** | **1** | **1** | **1** | **1** | **1** |
| **SLC1A4** | **hsa-miR-506-3p** | **1** | **1** | **1** | **0** | **0** |
| **SLC1A4** | **hsa-miR-214-3p** | **1** | **1** | **1** | **0** | **0** |
| **SLC1A4** | **hsa-miR-761-3p** | **1** | **1** | **1** | **0** | **0** |
| **SLC1A4** | **hsa-miR-3619-5p** | **1** | **1** | **1** | **0** | **0** |
| **SLC1A4** | **hsa-miR-145-5p** | **0** | **1** | **1** | **0** | **1** |
| **SCP2** | **hsa-miR-1224-5p** | **1** | **1** | **1** | **0** | **0** |
| **SCP2** | **hsa-miR-3612** | **1** | **1** | **1** | **0** | **0** |
| **SCP2** | **hsa-miR-761** | **1** | **1** | **1** | **0** | **0** |
| **MUC1** | **hsa-miR-485-5p** | **1** | **1** | **1** | **0** | **1** |
| **TF** | **hsa-miR-519a-3p** | **1** | **1** | **1** | **0** | **0** |
