# Supplementary material for: Bioinformatics analysis of ferroptosis-related genes and immune cell infiltration in non-alcoholic fatty liver disease
Source: Eur J Med Res. 2023 Dec 19;28:605. doi: 10.1186/s40001-023-01457-0 (PMC10729346; doi:10.1186/s40001-023-01457-0)
Supplement: Supplementary file 6 — Additional file 6: Table S6. miRNAs interact with lincRNAs. [file 40001_2023_1457_MOESM6_ESM.docx]

**Table S6 . miRNAs interact with lincRNAs**

| **miRNAname** | **geneName** | **geneType** | **chromosome** | **start** | **end** | **clipExpNum** |
| --- | --- | --- | --- | --- | --- | --- |
| **hsa-miR-1270-3p** | **MALAT1** | **lincRNA** | **chr11** | **65273453** | **65273475** | **28** |
| **hsa-miR-1270-3p** | **AP000233.2** | **lincRNA** | **chr21** | **26473470** | **26473494** | **22** |
| **hsa-miR-1270-3p** | **AC245033.4** | **lincRNA** | **chr15** | **83207680** | **83207702** | **16** |
| **hsa-miR-1270-3p** | **SNHG8** | **lincRNA** | **chr4** | **119200328** | **119200350** | **14** |
| **hsa-let-7e-5p** | **NUTM2A-AS1** | **lincRNA** | **chr10** | **89005067** | **89005093** | **20** |
| **hsa-let-7e-5p** | **XIST** | **lincRNA** | **chrX** | **73046461** | **73046481** | **19** |
| **hsa-let-7e-5p** | **NEAT1** | **lincRNA** | **chr11** | **65205185** | **65205207** | **17** |
| **hsa-let-7e-5p** | **LINC02381** | **lincRNA** | **chr12** | **54526512** | **54526534** | **16** |
| **hsa-miR-506-3p** | **MALAT1** | **lincRNA** | **chr11** | **65273028** | **65273048** | **27** |
| **hsa-miR-506-3p** | **FGD5-AS1** | **lincRNA** | **chr3** | **14984996** | **14985018** | **19** |
| **hsa-miR-506-3p** | **NEAT1** | **lincRNA** | **chr11** | **65193519** | **65193539** | **17** |
| **hsa-miR-214-3p** | **LINC00665** | **lincRNA** | **chr19** | **36811245** | **36811263** | **14** |
| **hsa-miR-214-3p** | **NEAT1** | **lincRNA** | **chr11** | **65205579** | **65205600** | **12** |
| **hsa-miR-761-3p** | **LINC00665** | **lincRNA** | **chr19** | **36811245** | **36811268** | **14** |
| **hsa-miR-761-3p** | **NEAT1** | **lincRNA** | **chr11** | **65205579** | **65205600** | **12** |
| **hsa-miR-3619-5p** | **LINC00665** | **lincRNA** | **chr19** | **36811245** | **36811265** | **14** |
| **hsa-miR-3619-5p** | **NEAT1** | **lincRNA** | **chr11** | **65205577** | **65205600** | **12** |
| **hsa-miR-145-5p** | **SNHG1** | **lincRNA** | **chr11** | **62621371** | **62621392** | **16** |
| **hsa-miR-145-5p** | **AC023509.1** | **lincRNA** | **chr12** | **53849761** | **53849782** | **13** |
| **hsa-miR-145-5p** | **MALAT1** | **lincRNA** | **chr11** | **65267120** | **65267142** | **12** |
| **hsa-miR-1224-5p** | **NEAT1** | **lincRNA** | **chr11** | **65205206** | **65205225** | **19** |
| **hsa-miR-1224-5p** | **AC009133.5** | **lincRNA** | **chr16** | **29820019** | **29820037** | **12** |
| **hsa-miR-3612** | **MIR155HG** | **lincRNA** | **chr21** | **26946307** | **26946328** | **27** |
| **hsa-miR-3612** | **AL355075.4** | **lincRNA** | **chr14** | **20811341** | **20811362** | **24** |
| **hsa-miR-3612** | **MIR663AHG** | **lincRNA** | **chr20** | **26189810** | **26189825** | **24** |
| **hsa-miR-3612** | **AC068768.1** | **lincRNA** | **chr12** | **123745993** | **123746015** | **12** |
| **hsa-miR-3612** | **NEAT1** | **lincRNA** | **chr11** | **65195462** | **65195483** | **10** |
| **hsa-miR-485-5p** | **MALAT1** | **lincRNA** | **chr11** | **65272750** | **65272771** | **30** |
| **hsa-miR-485-5p** | **AC005899.4** | **lincRNA** | **chr17** | **30687656** | **30687681** | **24** |
| **hsa-miR-485-5p** | **NEAT1** | **lincRNA** | **chr11** | **65198512** | **65198534** | **15** |
| **hsa-miR-485-5p** | **MIR29B2CHG** | **lincRNA** | **chr1** | **207975182** | **207975203** | **13** |
| **hsa-miR-485-5p** | **AC016876.2** | **lincRNA** | **chr17** | **7482121** | **7482140** | **13** |
| **hsa-miR-519a-3p** | **MIR17HG** | **lincRNA** | **chr13** | **92003602** | **92003625** | **47** |
| **hsa-miR-519a-3p** | **AC021078.1** | **lincRNA** | **chr5** | **148874435** | **148874456** | **35** |
| **hsa-miR-519a-3p** | **AC010542.4** | **lincRNA** | **chr16** | **66584438** | **66584459** | **33** |
| **hsa-miR-519a-3p** | **NEAT1** | **lincRNA** | **chr11** | **65204977** | **65204998** | **18** |
| **hsa-miR-519a-3p** | **XIST** | **lincRNA** | **chrX** | **73042795** | **73042818** | **18** |
| **hsa-miR-519a-3p** | **H19** | **lincRNA** | **chr11** | **2017147** | **2017168** | **15** |
| **hsa-miR-519a-3p** | **AC021092.1** | **lincRNA** | **chr19** | **44612231** | **44612252** | **15** |
| **hsa-miR-519a-3p** | **AC243964.3** | **lincRNA** | **chr19** | **45167433** | **45167454** | **15** |
| **hsa-miR-519a-3p** | **AL158206.1** | **lincRNA** | **chr9** | **19454928** | **19454955** | **13** |
| **hsa-miR-519a-3p** | **EPB41L4A-AS1** | **lincRNA** | **chr5** | **111497987** | **111498008** | **12** |
