# Supplementary material for: Bioinformatics analysis of ferroptosis-related genes and immune cell infiltration in non-alcoholic fatty liver disease
Source: Eur J Med Res. 2023 Dec 19;28:605. doi: 10.1186/s40001-023-01457-0 (PMC10729346; doi:10.1186/s40001-023-01457-0)
Supplement: Supplementary file 7 — Additional file 7: Table S7. miRNAs interact with circRNAs. [file 40001_2023_1457_MOESM7_ESM.docx]

**Table S7 . miRNAs interact with circRNAs**

| **miRNAname** | **geneName** | **geneType** | **chromosome** | **start** | **end** | **clipExpNum** |
| --- | --- | --- | --- | --- | --- | --- |
| **hsa-miR-1270-3p** | **GARS** | **circRNA** | **chr7** | **30656786** | **30656808** | **50** |
| **hsa-miR-1270-3p** | **ZNFX1** | **circRNA** | **chr20** | **47863306** | **47863325** | **49** |
| **hsa-let-7e-5p** | **ACTG1** | **circRNA** | **chr17** | **79478448** | **79478469** | **53** |
| **hsa-let-7e-5p** | **GARS** | **circRNA** | **chr7** | **30656768** | **30656787** | **52** |
| **hsa-let-7e-5p** | **ACTB** | **circRNA** | **chr7** | **5568146** | **5568167** | **49** |
| **hsa-let-7e-5p** | **BACH1** | **circRNA** | **chr21** | **30717420** | **30717442** | **46** |
| **hsa-let-7e-5p** | **TAF5L** | **circRNA** | **chr1** | **229738376** | **229738397** | **42** |
| **hsa-let-7e-5p** | **ABT1** | **circRNA** | **chr6** | **26598906** | **26598928** | **41** |
| **hsa-let-7e-5p** | **NUPL1** | **circRNA** | **chr13** | **25887102** | **25887124** | **41** |
| **hsa-miR-506-3p** | **VAMP3** | **circRNA** | **chr1** | **7839894** | **7839914** | **38** |
| **hsa-miR-214-3p** | **CD151** | **circRNA** | **chr11** | **836110** | **836130** | **45** |
| **hsa-miR-214-3p** | **MAPK1** | **circRNA** | **chr22** | **22160162** | **22160181** | **44** |
| **hsa-miR-214-3p** | **TBL1XR1** | **circRNA** | **chr3** | **176741969** | **176741987** | **41** |
| **hsa-miR-214-3p** | **HDGF** | **circRNA** | **chr1** | **156713199** | **156713220** | **37** |
| **hsa-miR-761-3p** | **CD151** | **circRNA** | **chr11** | **836106** | **836130** | **45** |
| **hsa-miR-761-3p** | **MAPK1** | **circRNA** | **chr22** | **22160162** | **22160183** | **44** |
| **hsa-miR-761-3p** | **TBL1XR1** | **circRNA** | **chr3** | **176741969** | **176741990** | **41** |
| **hsa-miR-761-3p** | **HDGF** | **circRNA** | **chr1** | **156713199** | **156713220** | **37** |
| **hsa-miR-3619-5p** | **CD151** | **circRNA** | **chr11** | **836110** | **836130** | **45** |
| **hsa-miR-3619-5p** | **MAPK1** | **circRNA** | **chr22** | **22160162** | **22160183** | **44** |
| **hsa-miR-3619-5p** | **TBL1XR1** | **circRNA** | **chr3** | **176741969** | **176741993** | **41** |
| **hsa-miR-3619-5p** | **HDGF** | **circRNA** | **chr1** | **156713199** | **156713222** | **37** |
| **hsa-miR-145-5p** | **ACTG1** | **circRNA** | **chr17** | **79477613** | **79477635** | **50** |
| **hsa-miR-145-5p** | **NUP43** | **circRNA** | **chr17** | **79477613** | **79477635** | **50** |
| **hsa-miR-145-5p** | **CNOT6** | **circRNA** | **chr6** | **150047124** | **150047146** | **44** |
| **hsa-miR-1224-5p** | **HSPA8** | **circRNA** | **chr11** | **122929849** | **122929869** | **31** |
| **hsa-miR-3612** | **ATP6V0E1** | **circRNA** | **chr5** | **172461561** | **172461581** | **45** |
| **hsa-miR-485-5p** | **DDX17** | **circRNA** | **chr22** | **38882028** | **38882049** | **39** |
| **hsa-miR-485-5p** | **HSP90AB1** | **circRNA** | **chr6** | **44221384** | **44221404** | **36** |
| **hsa-miR-485-5p** | **ACTG1** | **circRNA** | **chr17** | **79477367** | **79477391** | **35** |
| **hsa-miR-519a-3p** | **MIDN** | **circRNA** | **chr19** | **1257711** | **1257732** | **63** |
| **hsa-miR-519a-3p** | **CLTC** | **circRNA** | **chr17** | **57746176** | **57746196** | **57** |
| **hsa-miR-519a-3p** | **LAPTM4A** | **circRNA** | **chr2** | **20232648** | **20232667** | **53** |
| **hsa-miR-519a-3p** | **UBC** | **circRNA** | **chr12** | **125396220** | **125396241** | **51** |
| **hsa-miR-519a-3p** | **MSMO1** | **circRNA** | **chr4** | **166264169** | **166264190** | **49** |
| **hsa-miR-519a-3p** | **ZNFX1** | **circRNA** | **chr20** | **47862474** | **47862495** | **49** |
| **hsa-miR-519a-3p** | **SHOC2** | **circRNA** | **chr10** | **112772703** | **112772726** | **47** |
| **hsa-miR-519a-3p** | **ATP6V0E1** | **circRNA** | **chr5** | **172461541** | **172461562** | **46** |
| **hsa-miR-519a-3p** | **UBE3C** | **circRNA** | **chr7** | **156974314** | **156974336** | **46** |
| **hsa-miR-519a-3p** | **HSPA8** | **circRNA** | **chr11** | **122928266** | **122928287** | **46** |
| **hsa-miR-519a-3p** | **circ_KLHL28** | **circRNA** | **chr14** | **45397975** | **45397996** | **46** |
